# When artificial intelligence meets protein research

**DOI:** 10.12688/openreseurope.20628.1

**Published:** 2025-07-15

**Authors:** Sonia Longhi, Salvador Ventura, Sandra Macedo-Ribeiro, Leandro G. Radusky, Jovana Kovačević, R. Gonzalo Parra, Miguel A. Andrade-Navarro, Andrey V. Kajava, Zuzana Bednáriková, Alexander Monzon, Rita Vilaça

**Affiliations:** 1 Aix Marseille University, Marseille, Provence-Alpes-Côte d'Azur, France; 2 Centre National de la Recherche Scientifique (CNRS), Lab. Architecture et Fonction des Macromolécules Biologiques (AFMB), Marseille, Provence-Alpes-Côte d'Azur, France; 3 Autonomous University of Barcelona Institute of Biotechnology and Biomedicine, Bellaterra, Catalonia, Spain; 4 Universitat Autonoma de Barcelona Departament de Bioquimica i Biologia Molecular, Bellaterra, Catalonia, Spain; 5 Institut d'Investigacio i Innovacio Parc Tauli, Sabadell (I3PT-CERCA), Catalonia, Spain; 6 Hospital Universitari Parc Taulí, Sabadell, Spain; 7 Universidade do Porto Instituto de Investigacao e Inovacao em Saude, Porto, Porto District, Portugal; 8 Mimark Diagnostics S.L., Parc Cientific Barcelona, Barcelona, Catalonia, Spain; 9 University of Belgrade Faculty of Mathematics, Belgrade, Serbia; 10 The Institute for Artificial Intelligence Research and Development of Serbia, Novi Sad, Serbia; 11 Life Sciences Department, Barcelona Supercomputing Center, Barcelona, Barcelona, Spain; 12 Johannes Gutenberg University Mainz Institute of Organismic and Molecular Evolution, Mainz, Rhineland-Palatinate, Germany; 13 Centre de Recherche en Biologie cellulaire de Montpellier, Université de Montpellier, CNRS, Montpellier, Montpellier, France; 14 Institute of Experimental Physics Slovak Academy of Sciences, Košice, Košice Region, Slovakia; 15 University of Padua Department of Biomedical Sciences, Padua, Veneto, Italy; 16 REQUIMTE LAQV Porto, Porto, Porto District, Portugal

**Keywords:** artificial intelligence, machine learning, non-globular proteins, deep learning, structural biology, bioinformatics, protein folding

## Abstract

The 2024 Nobel Prizes in Chemistry and Physics mark a watershed moment in the convergence of artificial intelligence (AI) and molecular biology. This article explores how AI, particularly deep learning and neural networks, has revolutionized protein science through breakthroughs in structure prediction and computational design. It highlights the contributions of 2024 Nobel laureates John Hopfield, Geoffrey Hinton, David Baker, Demis Hassabis, and John Jumper, whose foundational work laid the groundwork for AI tools such as AlphaFold. These tools are transforming our understanding of protein folding, and the dynamics of non-globular proteins, including intrinsically disordered proteins. While AI-driven methods have made predicting protein structures faster and more accessible, they also underscore ongoing scientific challenges, including the dynamics of protein folding and amyloid aggregation. European initiatives, such as the COST Actions NGP-net (BM1405) and ML4NGP (CA21160), are spearheading efforts to bridge these gaps by integrating AI and experimental data in the study of non-globular proteins. Together, these developments signal a transformative shift in biology, paving the way for novel discoveries in medicine, biotechnology, and materials science.

## Disclaimer

The views expressed in this article are those of the authors. Publication in Open Research Europe does not imply endorsement of the European Commission.

Artificial intelligence (AI) is revolutionizing biology, unlocking mysteries that defied previous understanding. The 2024 Nobel Prizes in Physics and Chemistry recognize this transformation, honoring researchers whose work has reshaped our understanding of biomolecules, including proteins, the molecular machines of life. From neural networks that decode complex biological data to AI-driven models capable of predicting protein structures with near-experimental accuracy, these breakthroughs are accelerating discoveries in medicine, biotechnology, and beyond. As we stand at the intersection of AI and molecular science, it is clear that we are witnessing the dawn of a new era in biology.

## Nobel laureates pioneering New Frontiers in protein science

The 2024 Nobel Prizes in Physics and Chemistry reflect the growing impact of AI in revolutionizing biological sciences, particularly in the field of protein structure prediction and *de novo* computational protein design. John Hopfield and Geoffrey Hinton were awarded the 2024 Nobel Prize in Physics for their foundational contributions to artificial neural networks, while the Nobel Prize in Chemistry was awarded to David Baker for his innovations in computational protein design and to Demis Hassabis and John M. Jumper for the development of AlphaFold, an AI system that predicts protein structures with unprecedented accuracy.

The fundamental scientific contributions of these laureates have catalysed advances in machine learning, bioinformatics, and structural biology, opening new avenues for understanding the complex behaviour of proteins, including the fascinating class of non-globular proteins. Together, these achievements offer extraordinary promise for building better working hypotheses to design experiments that advance our broader understanding of biology at the molecular level with implications in medical research, biotechnology and material sciences.

## From neural networks to protein science: the role of AI in biology

Hopfield and Hinton’s pioneering work on neural networks laid the foundation for today’s machine learning models, which have become integral tools for processing and interpreting vast amounts of experimental data. These models can now detect intricate patterns and generate predictions from complex datasets, an advancement crucial for fields like genomics, transcriptomics and proteomics. Their innovations in AI, particularly Hinton’s work on deep learning techniques and Hopfield’s neural network based on physics principles, have transformed how researchers analyse large-scale data, enabling them to uncover hidden patterns within complex biological data.

These combined contributions enhance our ability to analyse complex datasets with machine learning technologies but also reduce the need for manual intervention, making data interpretation more rapid, efficient and precise. “The 2024 Nobel Prize for Physics underscores how fundamental discoveries can have profound impacts across various scientific domains, including bioinformatics. This has revolutionised the study of biological processes, particularly in areas such as protein structure prediction” says Prof. Jovana Kovacevic.

## AlphaFold and the breakthrough in protein structure prediction

A major milestone in applying AI to biology was reached in 2021 when AlphaFold was developed by Demis Hassabis and John M. Jumper demonstrating an unprecedented ability to predict the **three-dimensional structure of proteins** from their amino acid sequence. This breakthrough, rooted in the principles of machine learning laid down by Hopfield and Hinton, has reshaped structural biology, giving access to structural data even in the absence of experimental data obtained through costly and time-consuming techniques. One must not forget that the impactful advancements in AI-based prediction of protein structures stand on the shoulders of experimental protein structure determination. The pioneering development of the Protein Data Bank (PDB), established in 1971, one of the first open-access databases for biological data, played a crucial role in enabling and expediting these breakthroughs in AI-based methodologies for structure prediction. The PDB set a precedent by creating a centralized repository of high-quality, expertly curated, three-dimensional atomic coordinates of proteins and nucleic acids, leveraging AI-driven predictors like AlphaFold to train models. “In the end, AI-based prediction models are improving and expediting experimental determinations of protein structures, being routinely used in the experimental structure determination workflows.” says Sandra Macedo-Ribeiro.

While AlphaFold has made significant strides in predicting globular protein structures, non-globular proteins (NGPs) pose unique challenges due to their irregular shapes and the disordered nature of regions thereof. NGPs, which are involved in numerous biological processes, have long defied conventional predictive methods due to their unique properties, such as high flexibility, lack of a predominant equilibrium conformation, and high conformational heterogeneity.

Deep learning models are making strides in predicting NGP functions, interactions, and roles in cellular processes. Neural networks are particularly promising in enhancing functional prediction accuracy and discovering complex protein-protein interactions. Just as physics expands our understanding of the universe, machine learning is helping decode the intricate world of proteins, especially those that defy conventional categorization.

“The availability of AlphaFold models and easy access to ColabFold and AlphaFold2 Colab servers for custom predictions, have contributed considerably to growing awareness of intrinsically disordered regions (IDRs) in proteins. Long “loopy” regions devoid of regular secondary structure, which are often predicted with low confidence in AlphaFold models, visually catch the attention of scientists. “says Sonia Longhi.

## Nobel-winning chemistry: computational protein design and AlphaFold

The Nobel Prize in Chemistry for David Baker highlights another key area where AI is pushing boundaries: *de novo* computational protein design. Baker’s research allows scientists to design synthetic proteins for applications in medicine, biotechnology, and materials science. This work complements the achievements of Hassabis and Jumper, who have demonstrated the predictive power of AI with AlphaFold, which is already making an impact in fields like drug discovery and enzyme engineering.

 For more than half a century, the “holy grail” of bioinformatics has been about how to decipher the information that is contained within a polypeptide to predict the 3D structure that it would acquire once in solvent, e.g. water.

Sequencing technologies allowed us to obtain the sequences of millions of proteins (approximately 200 million are contained in the UniProt database). However, experimentally solving their corresponding 3D structures would be an unimaginably expensive process, both in time and money. After developing some of the first algorithms aiming to solve the structure prediction problem, in 2003, David Baker produced the first *de novo* designed protein, which showed to be foldable after being expressed in the wet lab ( Basanta *et al.*, 2016). However, after some initial success, predicting the structures of complex proteins remained elusive. It was in 2021, two years after its first version, that the group led by John Jumper and Demis Hassabis published AlphaFold2. “This software was able to predict in minutes the structures of proteins that experimental labs have failed to solve for almost a decade. This was the beginning of an unprecedented revolution in molecular biology where the sequence/structure gap was closed and suddenly we started having access to good-quality predictions of structures of a majority of known proteins in the planet.” says Dr Gonzalo Parra.

The ability to predict the structure of proteins from their amino acid sequence has profound implications for biomedicine, biochemistry, and biotechnology. As AlphaFold continues to evolve, researchers across the globe are now applying its principles to tackle more complex and irregular protein structures, such as non-globular proteins and protein complexes. From drug design to building synthetic materials, the consequences of these developments are yet to be assessed. One thing is clear: the spotlight right now is on Computational Biology, and it will be so for a long time.

**The impact of AI on structural biology: a researcher’s perspective**

Dr. Leandro Radusky, a structural biologist, recalls the first time he encountered AlphaFold:

“The first time I seriously paid attention to AlphaFold was when the results of the 14th edition of the CASP protein structure prediction competition were announced in 2020. Like most people working in this field, my initial reaction was disbelief. The ability to predict structures from a protein’s sequence had made a massive leap, using a completely different approach from what had been employed for decades.” After AlphaFold’s release, the impact was immediate: “Shortly after the release of the tool’s code, the structure predictions of all human proteins and many other organisms of interest became publicly available. Overnight, the number of proteins with reasonably accurate structures available went from a few hundred thousand to millions.” Despite this, Radusky raises an important question about the balance between AI-driven methods and traditional scientific approaches:

“Beyond the technological leap, AlphaFold has highlighted a critical choice young researchers are facing today: how much should they rely on traditional interpretive science versus AI-driven approaches? With AI, we are often trading understanding for the ability to solve highly complex problems. As we move forward, the question seems not to be whether to use AI, but how to integrate it thoughtfully into the pursuit of understanding.”

## The future of AI in protein science

Without any doubt, AI-based tools like AlphaFold represent a breakthrough in structural biology, with extensive applications in protein structure prediction which deserved the Nobel Prize recognition. Yet, this does not signal the end of discoveries in this field, as many challenges in structural biology remain unresolved.

One major challenge is understanding the molecular mechanisms and atomic interactions that guide a polypeptide chain to fold into its specific three-dimensional structure. This has been a longstanding question in structural biology, originally attracting many talented physicists to the field. Despite significant advances, a comprehensive theory of protein folding based on physical principles that would offer a deep understanding of these processes is still missing. “AI-based methods, with their ability to generate reliable structural models, are poised to greatly advance our understanding of protein folding mechanisms. These remaining challenges offer exciting opportunities for groundbreaking advances in protein structure research, with the potential for future Nobel Prizes built on the achievements of AI-driven methods” says Prof. Andrey V. Kajava.

While researchers in the field of protein disorder are certainly excited by the progress brought by AlphaFold and the more recently developed AlphaFold Multimer, which extends predictions to protein complexes, one must remain aware of the current limits of these systems: they produce predictions that do not take into account the dynamic properties of protein structures and depend on experimentally solved structures, which might be affected by different experimental conditions and molecular partners. Concerning IDRs, AlphaFold appears to have a tendency to overpredict helical structures. Fortunately, AI-based methods are continuously being improved, and the community is advancing in developing new or improving existing AI-driven tools to specifically study intrinsically disordered proteins (IDPs). For instance, machine learning methods are already being used to learn force fields specific for molecular dynamics simulations of IDRs.

Another question arises when approaching complex biological systems, such as amyloid structures. Although biologically relevant as they are associated with debilitating neurodegenerative and other conformational diseases, these structures cannot yet be solved by AI-based approaches owing to their high complexity. Deciphering the amyloid structures formed during protein aggregation is challenging, as protein aggregation is not guided by functional constraints or evolutionary pressure, which typically help maintaining specific interactions. As a result, most of the aggregated species lack the residue coevolution patterns that AlphaFold and similar predictors depend on, creating a significant limitation for structure prediction in this context. Furthermore, the aggregation process generates a variety of structures—intermediates, oligomers, and amyloid fibrils—leading to a highly complex protein landscape. “It's becoming clear that the ability of a single polypeptide sequence to form multiple stable amyloid structures is not rare. Instead, it underscores that the aggregation process is controlled by kinetics rather than thermodynamics. This means that a single protein sequence can produce various stable forms, challenging Anfinsen's principle, which traditionally underpins AI-sequence-based predictions” says Prof. Salvador Ventura.

Nevertheless, these AI-based tools like AlphaFold are highly dependent on experimental data. The recent advances in the resolution of protein structures by electron cryo-EM, which has reached atomic resolution similar to X-ray crystallography in recent years, have revitalised the pace at which novel structures are deposited in databases, addressing the resolution of very large complexes that until recently were beyond the reach of experimental resolution. AI-driven methodologies are also becoming increasingly integrated into electron cryo-EM for resolving large protein complexes at atomic resolution. AI’s reliance on detecting protein contacts through correlated mutations has been significantly enhanced by the sequencing of complete genomes, making it possible to derive statistically significant structural information, considering that for many proteins, we have thousands of corresponding homologs computationally translated from the fully sequenced genomes as potential transcripts. “This alone justifies not only efforts to sequence as many organisms as possible but also conservationist initiatives across the planet, as each species’ genome contributes to our knowledge of protein variations, and human-made climate change challenges the existence of entire ecosystems.” says Prof. Miguel Andrade.

## AI as a transformative force in biology

The Nobel Prizes for 2024 mark a turning point for AI’s role in advancing complex dogmas in Biology like the protein sequence-structure-function prediction. The integration of Baker’s computational design with the foundational principles of AI laid down by Hopfield and Hinton, as well as Hassabis and Jumper’s AlphaFold, underscores the transformative potential of machine learning in solving complex biological problems. These innovations will not only deepen our knowledge of biological macromolecules, such as proteins, but will also enhance drug development and the treatment of diseases driven by protein misfolding and pathological aggregation, including neurodegenerative diseases and cancer. As these tools continue to evolve, the scientific community stands on the brink of uncovering new insights into non-globular proteins that are key to novel therapeutic approaches and a deeper understanding of life at the molecular level.

This impressive wealth of three-dimensional protein models significantly enhances awareness of the relevance of protein structure in biological research. While experimental validation remains necessary, they undoubtedly provide outstanding starting points not only for structural biologists aiming to elucidate experimental structures of proteins but also for other researchers studying the physiological and pathological implications of target proteins.

As new developments in AI methods continue to unfold, the scientific community is looking forward to witnessing advancements in predicting protein structural dynamics and conformational biases in IDPs, which represent the next challenge in the quest to decode protein function. In this regard, developing open-access databases with experimental data on protein conformational dynamics and ensembles will provide the foundations for robust high-quality AI-driven predictions.

The Nobel Prizes awarded last year highlight the extraordinary potential of integrating AI into biological research. By applying these AI-driven techniques to non-globular proteins, we now have the tools to solve some of the most challenging questions in biology.

## COST actions paving the way to integrate AI and disordered proteins

As we celebrate these groundbreaking achievements, it becomes increasingly clear that the integration of machine learning in protein science is not a passing trend but a transformative force that will shape the future of science for decades to come. While AlphaFold and similar AI models have revolutionized the prediction of globular protein structures, the complexity of non-globular proteins (NGPs) continues to pose unique challenges. Recognizing this gap, European scientific collaboration through COST Actions has played a pioneering role in shaping the research agenda around NGPs.

The COST Action BM1405: Non-globular proteins – from sequence to structure, function and application in molecular physiopathology (NGP-net) was instrumental in establishing a pan-European research community dedicated to exploring the complexity of NGPs. This Action united experimentalists, computational biologists, and medical researchers, creating a fertile ground for knowledge exchange and cross-disciplinary innovation. The action fostered new standards for disorder annotations, facilitated the sharing of experimental protocols, and catalyzed collaborations that led to key advances in our understanding of protein disorder and its role in disease mechanisms.

Building on these foundations, the COST Action CA21160: Non-globular proteins in the era of Machine Learning (ML4NGP), started in 2022, is driving a new paradigm that integrates machine learning and deep learning technologies to tackle the enduring challenges of NGPs. ML4NGP aligns directly with the scientific momentum recognized by the 2024 Nobel Prizes by emphasizing how AI can be purposefully adapted to understand disordered and dynamic protein systems. This Action is currently developing community benchmarks, curating protein intrinsic disorder datasets, and promoting the development of interpretable AI models tailored for flexible protein regions. By organizing training schools, workshops, and open-access initiatives, ML4NGP ensures that the next generation of researchers has the skills and knowledge to maximize the benefits of these powerful new tools.

Together, NGP-net and ML4NGP form a clear path from scientific understanding to real-world technological applications. Their work highlights that while AI breakthroughs like AlphaFold are impressive, we still need to push further to truly capture the complexity and flexibility of non-globular proteins. In doing so, they are not only contributing to the scientific advancements spotlighted by the Nobel Committee but also ensuring that non-globular proteins, long in the shadow of structured proteins, are finally receiving the attention and methodological innovation they deserve. The Nobel Committee's recognition of AI’s potential in advancing protein science is a timely reminder that the biggest challenges in biology may finally be within reach.

## Ethics and consent

Ethical approval and consent were not required for this study.

## Data Availability

No new data were created or analysed in this study. Data sharing is not applicable to this open letter as it only presents opinions and perspectives from the authors.

## References

[ref-1] Basanta B Chan KK Barth P : Introduction of a polar core into the *de novo* designed protein Top7. *Protein Sci.* 2016;25(7):1299–307. 10.1002/pro.2899 26873166 PMC4918430

