# Supplementary material for: When artificial intelligence meets protein research
Source: Open Res Eur. 2025 Jul 15;5:185. [Version 1] doi: 10.12688/openreseurope.20628.1 (PMC12413608; doi:10.12688/openreseurope.20628.1)
Supplement: Peer review history

# Reviewer report: Reviewer response for version 1

**DOI:** 10.21956/openreseurope.22318.r58288

**Published:** 2025-09-05

**Authors:** Marcelo C. R. Melo

**Affiliations:** 1 Biochemistry and Molecular Biology, Colorado State University College of Natural Sciences, Fort Collins, Colorado, USA

In this open letter, the authors accurately describe the transformative and long-lasting effects AI models will have (and already have had) on biological research, especially the “extraordinary promise” they offer to medicine, biotechnology, and beyond. However, I believe more evidence is needed to claim that such models have “reshaped our understanding of biomolecules.” As discussed in the letter (and in other recent publications), the protein structure prediction and the protein folding process are different problems. AI models have reliably outperformed previous structure prediction methods, but such models have yet to reveal a new fundamental principle of protein folding. The authors highlight that “Despite significant advances, a comprehensive theory of protein folding based on physical principles that would offer a deep understanding of these processes is still missing.” I would suggest a more careful tone as to avoid conflating the promise and hopes we have for AI methods, and the achievements they have actually already reached. As mentioned in the letter, over reliance on AI can to lead to “trading understanding for the ability to solve highly complex problems.”

 With that said, the authors highlight the impact AI models can have in a variety of research endeavors. Moreover, they also describe how their use has drawn attention to complicated, difficult to determine structures, and even intrinsically disordered regions of proteins. Importantly, the authors highlight the fundamental work done by the Protein Data Bank and the decades of structural biology work, without which the AI models discussed here would not exist. Besides the structural databases, I would add that the development of the first AlphaFold models also relied on sequence databases mentioned elsewhere in this open letter.

 While the overall tone of the letter is very positive (and I believe it should be given the potential applications and possibility of innovation AI brings to protein research), it is important to remember the reader that such models are far from perfect. At times, structures are still predicted with low accuracy and detail, and generative models still can fail to produce biologically relevant structures altogether. While their overall impact is obviously positive, results from AI models still require validation and curation, which are new problems that require research and methods development.

 The letter has a single citation, but makes claims regarding current research that could be supported by a few more citations. For instance, authors mention that “… machine learning methods are already being used to learn force fields specific for molecular dynamics simulations of IDRs.” This is a very important topic, both in the context of IDR dynamics and AI-based structure prediction, so one or more citations here would help support the argument made by the authors. Similar instances are mentions of AI integration into experimental structure determination, and drug development.

 Since the field of deep learning, and specifically the applications to biomolecules, moves at impressively fast pace, it is inevitable that any manuscript would age quickly. However, when authors mention AlphaFold Multimer, they could also raise more recent example of AlphaFold 3, which made a very sharp change from sequence-based contact matrix prediction to fully generative structure prediction, and improved how it predicts protein complexes that involve ligands and nucleic acids. The very recent publication of BioEmu also shows how AI-based structure prediction is trying to tackle the dynamical nature of proteins, and even with its own caveats, can provide a broader distribution of predicted structures.

 The focus on non globular proteins, their complex dynamics and functions, and how integration between experimental and computational research will drive this new field of research, are well supported in the letter. Authors also propose a path for workforce development and training, as well as for the development of new research directions integrating AI to protein science.

Where applicable, are recommendations and next steps explained clearly for others to follow? (Please consider whether others in the research community would be able to implement guidelines or recommendations and/or constructively engage in the debate)

Yes

Does the article adequately reference differing views and opinions?

Yes

Are all factual statements correct, and are statements and arguments made adequately supported by citations?

Partly

Is the rationale for the Open Letter provided in sufficient detail? (Please consider whether existing challenges in the field are outlined clearly and whether the purpose of the letter is explained)

Yes

Is the Open Letter written in accessible language? (Please consider whether all subject-specific terms, concepts and abbreviations are explained)

Yes

Reviewer Expertise:

Computational biophysics; Protein dynamics

I confirm that I have read this submission and believe that I have an appropriate level of expertise to confirm that it is of an acceptable scientific standard.

# Reviewer report: Reviewer response for version 1

**DOI:** 10.21956/openreseurope.22318.r57519

**Published:** 2025-09-05

**Authors:** Judith Bernett, Konstantin Pelz

**Affiliations:** 1 TUM School of Life Sciences, Technical University of Munich, Freising, Bavaria, Germany

**Summary of the article**

 In this open letter, the authors present how computational biology receives more public spotlight, as in 2024, the physics Nobel prize was awarded for the invention of artificial neural networks, and the chemistry Nobel prize was awarded for computational protein design and protein structure prediction. They highlight current limitations of AI-driven protein research, particularly in predicting intrinsically disordered proteins, non-globular proteins, and amyloid structures. They finally present their COST actions on non-globular proteins.

 While the general topic of the article is timely and interesting to the general research community, we think it would benefit from some revisions. The article is repetitive and too long, given its relatively low information density. The text may be difficult for non-computational biologists to follow, as key concepts are not explained clearly. Moreover, the transitions between sections are weak, and the overall narrative flow requires improvement. Finally, we find the format of the letter with the direct quotes by the authors somewhat confusing.

**(1) Is the rationale for the Open Letter provided in sufficient detail? (Please consider whether existing challenges in the field are outlined clearly and whether the purpose of the letter is explained.)**

Yes.

**Justification: **The purpose of the letter is explained - AI is increasingly important in protein research and can successfully be applied, as demonstrated by the Nobel-awarded AlphaFold. 

**(2) Does the article adequately reference differing views and opinions?**

No.

**Justification: **The opinions stated are the opinions of the authors, sometimes with direct quotes, but the opinions do not differ much. Other opinion pieces are not mentioned or quoted.

**Recommendations: **It might make sense to include some more critical voices about the excessive AI usage, lack of interpretability, excessive use of energy, and untrustworthiness. Moreover, developing breakthrough AI models such as AlphaFold, Enformer, AlphaGenome, or ESM-2 lies beyond the scope of most research labs due to their limited computational and human resources.

**(3) Are all factual statements correct, and are statements and arguments made adequately supported by citations?**

Partly.

**Justification: **There is only one cited source, and the article contains bold, overexaggerated, and generalizing statements. Examples of the latter are 

- *“[AI is] unlocking mysteries that defied previous understanding”, *
- *“ Just as physics expands our understanding of the universe, machine learning is helping decode the intricate world of proteins.”, *
- *“One this is clear: the spotlight right now is on Computational Biology and it will be so for a long time”, *
- *“AI-based methods […] are poised to greatly advance our understanding of protein folding mechanisms”, *
- *“These innovations will not only deepen our knowledge on biological macromolecules […] but will also enhance drug development and the treatment of diseases”, *
- *“[…] the biggest challenges in biology may finally be within reach”. *

Statements that could use sources include:

- NGPs are challenging to predict (for AlphaFold)
- NGPs are involved in numerous biological processes
- *“[…] machine learning methods are already being used to learn force fields specific for molecular dynamics simulations of IDRs”*
- *“The action […] led to key advances in our understanding of protein disorder and its role […]”*
- Generally, the PDB, AlphaFold, ColabFold, and AlphaFold Multimer should be cited, as well as sources for the definition of NGPs, IDPs/IDRs, and amyloid structures.

**Recommendations: **Please insert missing sources and tone down these statements. The recent AI models are impressive, but they are still far from understanding the underlying biology, and we have no certainty about what the future will hold.

**(4) Is the Open Letter written in accessible language? (Please consider whether all subject-specific terms, concepts, and abbreviations are explained)**

Partly.

**Justification: **The language is accessible and the article is easy to read, but key subject-specific terms and concepts are not explained.

**Recommendations:** It might be useful to add a glossary explaining key concepts like non-globular proteins, intrinsically disordered regions/proteins, amyloid structures, and Anfinsen's principle, making the article accessible to people outside of the fields of bioinformatics and biology.

**(5) Where applicable, are recommendations and next steps explained clearly for others to follow? (Please consider whether others in the research community would be able to implement guidelines or recommendations and/or constructively engage in the debate)**

Partly.

**Justification: **The authors explain how research on non-globular proteins is still needed, which they address(ed) with their COST actions. However, this is not the only field of protein research that could still profit from AI models. Further, as previously pointed out, the article is mostly one-sided about applying AI to biology; it does not suggest usage guidelines or spark a debate.

**Recommendations:** It might be useful to add some caveats and some more critical points to stimulate constructive debate within the research community.

**(6) Other comments and recommendations**

1. The format of the letter is confusing to us. Eight of the eleven authors are directly quoted - does this mean the remaining three conducted interviews with them, making the piece resemble a feature article?
**Recommendation:** If this is indeed the case, it would be helpful to state it explicitly. If not, we would suggest removing the direct quotations and instead integrating these statements into the main text, since any content not otherwise attributed can reasonably be understood as representing the authors’ own views. 
2. The article contains repetitive parts, while not going into enough detail for more complicated concepts. The amazing powers of AI are repeatedly but vaguely stated without examples ( *AI catalysed advances in machine learning, bioinformatics, and structural biology; AI uncovers hidden patterns; AI is decoding the world of proteins; AlphaFold has profound implications for biomedicine, biochemistry, and biotechnology; AI advances complex problems in biology; with AI, we can solve some of the most challenging questions in biology; AI is a transformative source*). 
**Recommendation: **It would be helpful to provide some examples for the transformative powers of AI that are understandable to people outside the field of bioinformatics. What exactly can or was done now that was not possible before? Which concrete success stories were there? Then, these repeated sections could be condensed into one paragraph, freeing up some space to go into more details for, e.g., the glossary.
3. The transition from the Nobel prizes of 2024 to non-globular proteins, intrinsically disordered proteins, and the COST actions is not made very smoothly. Non-globular and intrinsically disordered proteins and amyloid aggregation are mentioned at different places throughout the text, but not followed up upon directly. 
**Recommendation:** Instead of just repeatedly mentioning NGPs, it could be beneficial to dedicate a paragraph or info box to them: what is this class of proteins, why are they hard to determine experimentally and, hence, harder to predict, and why are they important to study? Then, the transition to the COST actions would become smoother, too. This information could also be worked into an explicit paragraph about limitations of AlphaFold where the concept of intrinsically disordered regions and amyloid structures could be elaborated, too.
4. There are some minor semantical/grammatical/formal issues:
  1. *“In the end, AI-based prediction models are improving […|, being routinely used in […] workflows”*: This sentence is not grammatically correct.
  2. *“[…] and the disordered nature of regions thereof” *→ some of their regions?
  3. *“ **the three-dimensional structure of proteins**”* is the only bold part of the text - please remove this.
  4. *“The impressive wealth of three-dimensional protein models significantly enhances awareness of the relevance of protein structure in biological research.”*: This statement does not make sense.
  5. *“says Prof. Miguel Andrade”* - in the author list, the surname is stated as Andrade-Navarro.
  6. Make sure that the abbreviations are introduced the first time the long form is mentioned. This is, e.g., not the case for NGPs.

Where applicable, are recommendations and next steps explained clearly for others to follow? (Please consider whether others in the research community would be able to implement guidelines or recommendations and/or constructively engage in the debate)

Partly

Does the article adequately reference differing views and opinions?

No

Are all factual statements correct, and are statements and arguments made adequately supported by citations?

Partly

Is the rationale for the Open Letter provided in sufficient detail? (Please consider whether existing challenges in the field are outlined clearly and whether the purpose of the letter is explained)

Yes

Is the Open Letter written in accessible language? (Please consider whether all subject-specific terms, concepts and abbreviations are explained)

Partly

Reviewer Expertise:

Bioinformatics, computational biology, machine learning, implications of data leakage, protein-protein interaction prediction, alternative splicing, drug response prediction, cell-type deconvolution

We confirm that we have read this submission and believe that we have an appropriate level of expertise to confirm that it is of an acceptable scientific standard, however we have significant reservations, as outlined above.

# Reviewer report: Reviewer response for version 1

**DOI:** 10.21956/openreseurope.22318.r57516

**Published:** 2025-08-27

**Authors:** Nicola Bordin

**Affiliations:** 1 University College London, London, UK

In their open letter, Longhi and colleagues outline the dramatic impact artificial intelligence had on the fields of protein structure and computational biology, leading to long-lasting implications on research in the larger field of molecular biology. 

 I agree with all opinions on both merits and limitations of the tools highlighted, particularly the need for further research on non-globular proteins, amyloids and the process of folding. 

 If the authors are not constrained by word limits, this letter would benefit by mentioning another key factor that changed the field of computational biology concurrently with AlphaFold and protein design: protein language models. pLMs, in conjunction with these novel structure-based predictors are powering the research behind the next ten years of discoveries and should be mentioned as a key aspect of the "ML" part of "ML4NGP". 

 I have no further comments.

Where applicable, are recommendations and next steps explained clearly for others to follow? (Please consider whether others in the research community would be able to implement guidelines or recommendations and/or constructively engage in the debate)

Not applicable

Does the article adequately reference differing views and opinions?

Yes

Are all factual statements correct, and are statements and arguments made adequately supported by citations?

Yes

Is the rationale for the Open Letter provided in sufficient detail? (Please consider whether existing challenges in the field are outlined clearly and whether the purpose of the letter is explained)

Yes

Is the Open Letter written in accessible language? (Please consider whether all subject-specific terms, concepts and abbreviations are explained)

Yes

Reviewer Expertise:

Protein Bioinformatics, AI, Globular Proteins, algorithm design for protein structure, sequence and function.

I confirm that I have read this submission and believe that I have an appropriate level of expertise to confirm that it is of an acceptable scientific standard.
